# Age, strain, and gut section shape the microbiome of commercial laying hens

**DOI:** 10.1016/j.psj.2026.107152

**Published:** 2026-05-21

**Authors:** Timur Yergalyiev, Christoph Roth, Markus Rodehutscord, Jana Seifert, Amélia Camarinha-Silva

**Affiliations:** a Institute of Animal Science, University of Hohenheim, Stuttgart, Germany; b HoLMiR - Hohenheim Center for Livestock Microbiome Research, University of Hohenheim, Stuttgart, Germany

**Keywords:** Laying hens, Inositol phosphate metabolism, Gut microbiome, 16S rRNA gene amplicon sequencing, Metagenomics

## Abstract

Gut microbiota, among other factors, may influence the overall performance of laying hens. To investigate how host genetics and age shape microbial communities, we profiled the gut microbiome of two commercial laying hen strains, Lohmann Brown-Classic and Lohmann LSL-Classic, across five anatomical sections (crop, gizzard, duodenum, ileum, caeca) at five ages spanning pullet development through late lay (10, 16, 24, 30, 60 weeks of age). We extracted RNA from the luminal content and performed 16S rRNA gene amplicon sequencing based on complementary DNA. Both strain and age had highly significant effects on community composition. The greatest shifts occurred between early development (10 weeks) and the onset of lay (16–24 weeks). To link taxa to function, we applied shotgun metagenomics to samples taken at 16 and 24 weeks, revealing strain-specific changes in functional profiles associated with the transition into egg production. We identified three groups of bacterial species that increased in abundance during the transition: lactic-acid producers (such as *Lactococcus raffinolactis, Ligilactobacillus aviarius, Lactobacillus pontis, etc.*), potential probiotic bacteria (*Megasphaera stantonii,​​ Megamonas funiformis, ​​Phocaeicola ​​coprophilus,* etc.), and opportunistic or egg-associated pathogens (*Comamonas ​testosteroni,​ ​Aeromonas ​caviae,​ ​Acinetobacter ​johnsonii*, etc.). Corresponding shifts were also observed in the functional profiles of inositol phosphate metabolism. Moreover, MAG-based analyses reported two bacterial species - *Gallibacterium ​​anatis​ * and ​​*Megamonas ​​hypermegale*, to contain high numbers of myoinositol-related genes. Together, our results demonstrate that genetic background and production phase both drive dynamic, section-specific changes in the gut microbiome of laying hens.

## Introduction

Laying hens undergo several functional shifts throughout their productive lifespan. Associated alterations in molecular pathways influence the aging process and overall health. Moreover, exposure to various environmental factors influences the interplay among the aforementioned processes, resulting in additional physiological changes (Keshavarz and Nakajima, 1993; Hurwitz et al., 1995; Proszkowiec-Weglarz et al., 2019). Therefore, to meet changing metabolic demands, especially the heightened requirements for calcium (Ca) and phosphorus (P) during skeletal growth and eggshell formation, diet composition is adjusted at key life stages; otherwise, an imbalanced diet might affect animal health and productivity (Ballou et al., 2016; Sinclair-Black et al., 2023).

During the post-hatch period, the gastrointestinal tract (GIT) undergoes rapid morphological and functional maturation to accommodate solid feed (Noy et al., 2011; Yegani and Korver, 2008). By 16 weeks of age, hens reach reproductive maturity, and egg laying typically initiates shortly thereafter (Shi et al., 2020; Yaxiao et al., 2025). Between 16 and 24 weeks, egg production rapidly increases to approximately 88% of peak output, coinciding with a dietary shift from grower to pre-layer/layer rations, characterized by increased Ca supplementation and significant oviduct development (Robinson et al., 2001; Rahman et al., 2018). After week 30, hens achieve peak laying performance; thereafter, egg production gradually declines toward 60 weeks of age (Huneau-Salaün et al., 2011).

This transition to laying eggs (16–24 weeks) imposes substantial physiological stress as hens increase Ca retention in preparation for eggshell calcification (Keshavarz and Nakajima, 1993), while adequate P absorption remains vital for bone mineralization and reproductive health (Shafey et al., 1990; Hurwitz et al., 1995; Proszkowiec-Weglarz et al., 2019). Both minerals are predominantly absorbed in the small intestine (Proszkowiec-Weglarz et al., 2019), making gut function central to nutrient homeostasis and overall hen welfare.

Concurrently, the gut microbiome establishes and stabilizes from the day of hatching, shifting from a low initial diversity to a mature community structure within several weeks (Ballou et al., 2016). Although recent studies have characterized hen microbiota at specific ages, often focusing on caecal communities or epithelial adherent bacteria (Callaway et al., 2009; Nordentoft et al., 2011; Videnska et al., 2013, 2014; Cheng et al., 2025), comprehensive, longitudinal surveys across multiple GIT sections are lacking, despite each compartment’s unique role in digestion and immunity.

To address this gap, we compared the intestinal microbiota of two widely used commercial strains, Lohmann Brown-Classic (LB) and Lohmann LSL-Classic (LSL), across five gut sections (crop, gizzard, duodenum, ileum, caeca) and five ages spanning pullet development through late lay. We hypothesized that community composition and functionality vary with both host strain and age. Using 16S rRNA gene amplicon sequencing (further referred to as amplicon) across all ages, complemented by shotgun metagenomics (MG) at 16 and 24 weeks, we aimed to elucidate strain- and age-specific microbial dynamics in different gut sections of lying hens.

## Materials and methods

### Sample collection, nucleic acid extraction and sequencing

**Fig. 1:**
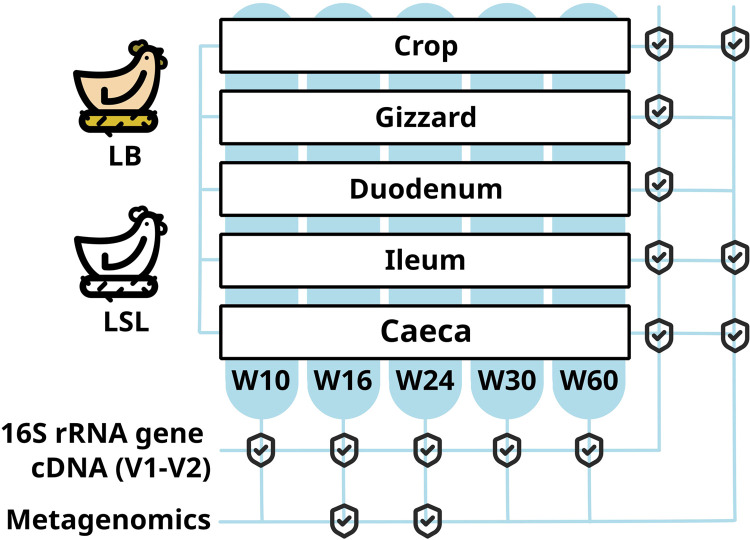
Graphical representation of the experimental setup.

A detailed description of the experimental setup is provided in Sommerfeld et al. (Sommerfeld et al., 2020). Briefly, 50 Lohmann Brown‐Classic (LB) and 50 Lohmann LSL‐Classic (LSL) hens, hatched together, were reared under identical conditions. The sampling scheme and experimental setup are represented in Fig. 1. Diets were based on corn and soybean meal, formulated to minimize intrinsic plant phytase activity, with composition adjusted to meet the nutritional requirements and feed‐intake levels specific to each age (10, 16, 24, 30, and 60 weeks of age, further referred to as W10, W16, W24, W30, and W60).

Until ten days before slaughter, all birds were kept on deep‐litter bedding. Then, 10 hens per strain were randomly selected and transferred to individual metabolic units (1 m³), arranged in a randomized block design to control for individual feed intake and metabolic data. During this time, the temperature in the animal house was maintained at 18–22°C; feed and tap water were available for *ad libitum* consumption. Two hours before slaughter, feed was removed and then reintroduced for one hour of ad libitum access before sampling, so that all hens were in a comparable short-term feeding state and variation in luminal content volume at slaughter was reduced.

On each sampling day, the 20 selected hens (10 LB and 10 LSL) were stunned using a gas mixture (35% CO₂, 35% N₂, 30% O₂) and immediately decapitated. The GIT was opened longitudinally, and the luminal content was aseptically collected from crop, gizzard, from the mid-duodenum and mid-ileum, and from the blind ends of both ceca, which were selected as major functional compartments for storage, mechanical breakdown, digestion and absorption, and hindgut fermentation in hens. Samples were immediately transferred into sterile, nuclease‑free tubes prefilled with RNAlater and stored at −80°C until RNA extraction.

Total RNA was extracted using TRIzol (Invitrogen, Carlsbad, CA, USA) according to the manufacturer's instructions, which included an initial bead-beating step (30 s, 5.5 m/s) in a FastPrep instrument (MP Biomedicals, Santa Ana, CA, USA). Extracted RNA was treated with DNase (Invitrogen), and cDNA was synthesized using SuperScript III First-Strand Synthesis System (Invitrogen). RNA purity was assessed spectrophotometrically, and only samples with adequate yield for cDNA synthesis and A260/280 ratios within an acceptable range for TRIzol-extracted intestinal RNA (approximately 1.6–2.0) were used for downstream library preparation.

Targeted amplification of the V1-V2 regions of the 16S rRNA gene was performed in a three‐step PCR using PrimeSTAR® HS DNA Polymerase (TaKaRa, Beijing, China) as described by Roth et al. (Roth et al., 2022). The first two PCRs (each with a total volume of 25 µL) used 1 µL of cDNA template, 0.2 µM primers, and 0.5 U PrimeSTAR HS. The third PCR (50 µL total volume) amplified indexed libraries. Thermal cycling consisted of an initial denaturation at 95°C for 3 min, followed by either 10 cycles (pre‐ and first PCR) or 20 cycles (third PCR) of 98°C for 10 s (denaturation), 55°C for 10 s (annealing), and 72°C for 45 s (extension), with a final extension at 72°C for 2 min. PCR‑grade water was included as a negative control during library preparation and sequenced together with the samples to monitor for contamination from reagents and handling. PCR products were pooled by index, normalized, and purified using the SequalPrep Normalization Kit (Invitrogen), and sequenced (250 bp paired‐end) on an Illumina NovaSeq 6000 platform. We also performed shotgun metagenomic sequencing of DNA samples from crop, ileum, and caeca at weeks 16 and 24. DNA was extracted from luminal content using the FastDNA™ SPIN Kit for Soil (MP Biomedicals; Catalog No. 6560 200) per the manufacturer’s instructions. Shotgun libraries were prepared according to standard Illumina paired‑end metagenomic library preparation procedures and were sequenced as150 bp paired‐end reads on an Illumina NovaSeq 6000. In both the Amplicon and MG datasets, 10 biological replicates were sequenced for each combination of hen strain, age, and section, yielding 500 amplicon and 120 MG samples. During the analyses, 11 amplicon samples were discarded for low sequencing depth, and 15 MG samples were discarded for high host DNA content (>90%)”.

### Bioinformatics and statistical analysis

The Amplicon dataset was analysed with Qiime2 (qiime2-amplicon-2025.4) (Bolyen et al., 2019). Raw reads were demultiplexed with Sabre (https://github.com/najoshi/sabre). Primer sequences were removed with q2-cutadapt (Martin, 2011). Amplicon sequencing variant (ASVs) counts, and representative sequences were obtained with q2-DADA2 (Callahan et al., 2016). Sequences were classified by using the Bayesian classifier trained on the GTDB (v. 226) (Parks et al., 2020).

Metagenomic (MG) raw reads were quality-controlled, and those aligned by Bowtie2 (Langmead and Salzberg, 2012) to the host genome (GRCg6a, GCA_000002315.5) were removed. Samples with a high host DNA content (>80%) were excluded, leaving 105 out of 120 samples. Cleaned MG samples were then analysed with Qiime2 (qiime2-moshpit-2025.4) (Ziemski et al., 2025). Metagenome assemblies were created for each sample using SPAdes (Bankevich et al., 2012) and assessed with QUAST (Gurevich et al., 2013). Binning was performed using COMEbin (Wang et al., 2024). BUSCO was used to evaluate the bins' "completeness" (the percentage of detected single-copy genes relative to their expected count) and "contamination" (the percentage of duplicated single-copy genes) (Simão et al., 2015). Only bins with ≥50% completeness and <20% contamination were dereplicated across the entire MG dataset and referred to as metagenome assembled genomes MAGs. Taxonomy was assigned to both MG reads and MAGs using Kraken2 (Wood et al., 2019) with the “pluspf” database, setting the confidence threshold to 0.1. Taxa abundances were estimated with Bracken2 (Lu et al., 2017). Functional annotations were performed with EggNOG mapper (Cantalapiedra et al., 2021) on MAGs using HMMER (Eddy, 2022), DIAMOND (Buchfink et al., 2015), and converted into Kyoto Encyclopedia of Genes and Genomes (KEGG) Orthology (further referred to as “KO”) (Kanehisa et al., 2025). High-quality MAGs were defined as those with ≥ 90% “completeness” and < 5% “contamination”.

For both Amplicon and MG samples, alpha diversity was assessed using Shannon’s entropy (Shannon, 2001), and beta diversity using robust Aitchison distances with CLR transformation (RPCA) (Martino et al., 2019). The differences in microbial alpha and beta diversity were tested with Kruskal-Wallis (Kruskal et al., 1952) and Adonis (Anderson, 2001) tests. P-values from multiple comparisons were adjusted with the Benjamin-Hochberg method (Benjamini and Hochberg, 1995). Differentially abundant taxa (at genus level for amplicon samples and at species level for MS) were detected with Ancom-BC2 (Lin et al., 2023).

## Results

### Microbiota diversity and composition

**Fig. 2:**
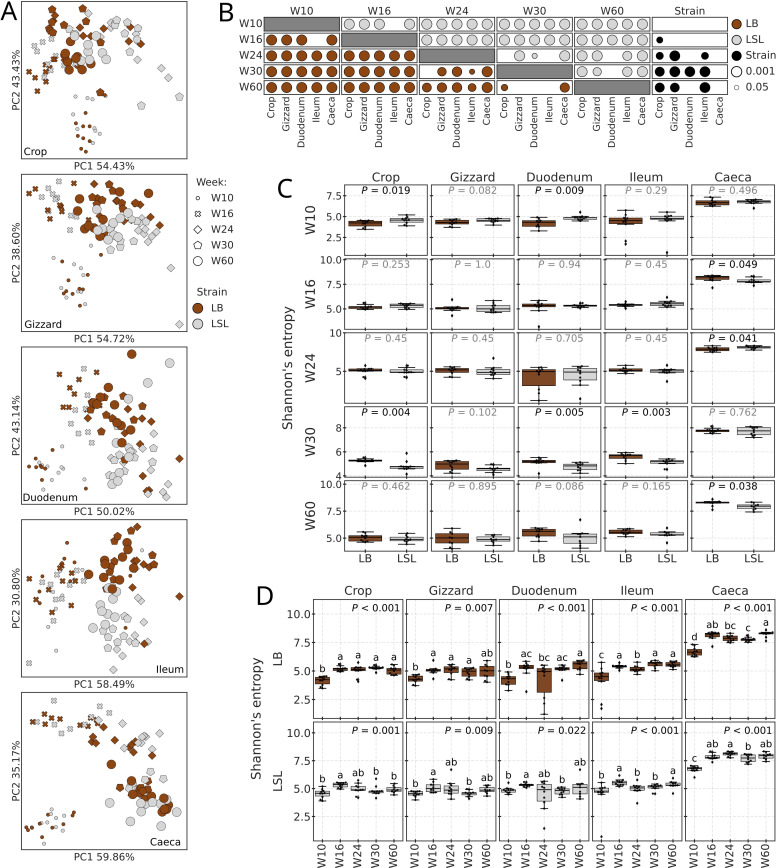
Amplicon dataset diversity and composition. A) PCoA plots by section based on RPCA distances. Weeks differentiated by marker shape and size, and strain by color. B) P-values (P-adjusted values when applicable) of between-weeks or strain comparisons for each section. Markers representing P-values/-adj of between-weeks comparisons for LB samples are colored in brown and LSL in light grey, while black markers indicate strain comparisons. Only significant P-values/-adj are shown (< 0.05), with bigger markers for smaller values. C) Alpha diversity (Shannon’s entropy) boxplots and Kruskal-Wallis test results when comparing strains by week and section. P-values of LB and LSL comparisons are plotted above boxplots in the center. D) Alpha diversity (Shannon’s entropy) boxplots and Kruskal-Wallis test results when comparing weeks by strain and section. P-values of the general test are located in the right corner at the top of each subplot, while pairwise comparisons based on the adjusted p-values are shown with compact letter display (P-adj < 0.05 for groups with different letters and P-adj ≥ 0.05 for groups with shared letters). For boxplots in subplots C and D, the line inside the box represents the median, while the whiskers represent the lowest and highest values (excluding outliers) and interquartile range (IQR).

Independent of the GIT section, the bacterial composition of the amplicon dataset was driven by both the age (W10-W60) and the host strain (Fig. 2A), with W10, in general, showing the greatest separation from the other weeks. Pairwise Adonis comparisons revealed that composition varied across ages for both strains, yielding mostly significant P-values after correction for multiple comparisons (Fig. 2B). The same test detected no differences between strains at W10 across all sections and in caecal samples from all weeks (all P-adj > 0.05). At W16, only composition in crop luminal content differed between strains (P-adj = 0.04), with no differences in other sections. In the duodenum, a significant difference was detected only at W30 (P-adj = 0.005). In crop, gizzard, and ileum, beta diversity differed between strains at ages W24, W30, and W60 (all P-adj ≥ 0.05). Shannon’s entropy of the same dataset was not affected by the strain in gizzard samples (Fig. 2C). In crop and duodenum, significant differences were detected at W10 (P-adj ≤ 0.019) and W30 (P-adj ≤ 0.005). In ileum, alpha diversity differed only at W30 (P-adj = 0.003). In caecal samples, unlike beta diversity, which demonstrated no differences between strains at each age, Shannon’s entropy was different at W16 (P-adj = 0.049), W24 (P-adj = 0.041), and W60 (P-adj = 0.038). In both strains and all sections, Shannon’s entropy was significantly affected by the week (all P-values ≤ 0.022) (Fig. 2D).

**Fig. 3:**
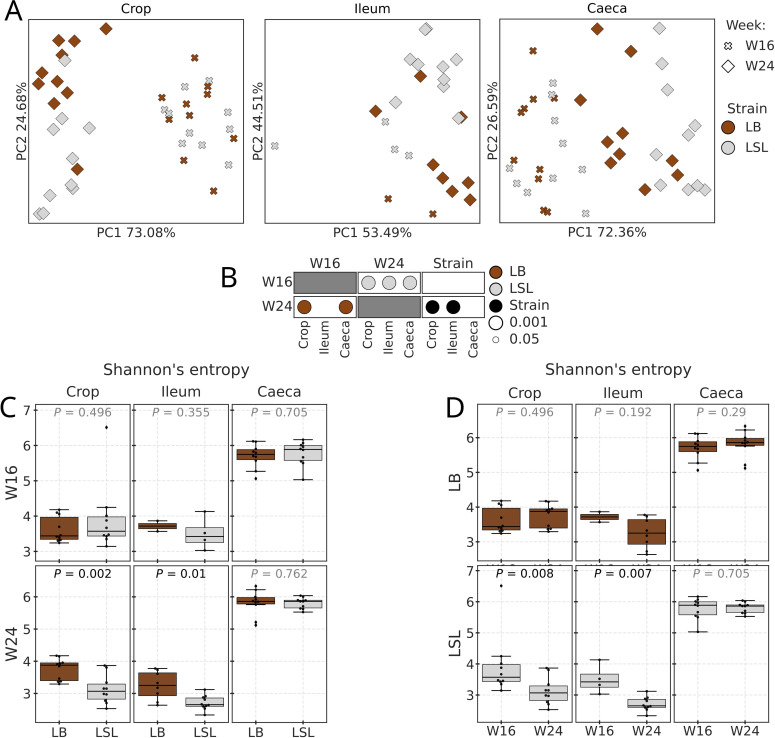
MG dataset diversity and composition. A) PCoA plots by section based on RPCA distances. Weeks are differentiated by marker shape and size, and strains by color. B) P-values (P-adjusted values when applicable) of between-weeks or strain comparisons for each section. Markers representing P-values/-adj of between-weeks comparisons for LB samples are colored in brown and LSL in light grey, while black markers indicate strain comparisons. Only significant P-values/-adj are shown (< 0.05), with bigger markers for smaller values. C) Alpha diversity (Shannon’s entropy) boxplots and Kruskal-Wallis test results when comparing strains by week and section. P-values of LB and LSL comparisons are plotted above boxplots in the center. D) Alpha diversity (Shannon’s entropy) boxplots and Kruskal-Wallis test results when comparing weeks by strain and section. P-values of W16 and W24 comparisons are plotted above the boxplots in the center. For boxplots in subplots C and D, the line inside the box represents the median, while the whiskers represent the lowest and highest values (excluding outliers) and interquartile range (IQR).

Prior analyses of data obtained from these hens identified the interval between weeks 16 and 24 as a distinct transition, marked by the onset of sexual maturity, oviduct development, and initiation of egg production (Sommerfeld et al., 2020; Gonzalez-Uarquin et al., 2021; Ponsuksili et al., 2021, 2023; Omotoso et al., 2021; Schmucker et al., 2021; Dreyling and Hasselmann, 2022). Accordingly, we focused our MG analysis on these two ages (W16 and W24), sampling from the crop, ileum and caeca, which were selected based on taxonomy profiles from amplicon dataset and the literature as sections in which microbiota plays an important role in egg laying associated processes (Ricke et al., 2022; Shi et al., 2024; Nafady et al., 2025). Similar to amplicon samples, the bacterial composition of MG dataset within each sampled GIT section was shaped by both the age and strain (Fig. 3A). Adonis test showed that ages W16 and W24 were different in each section of the LSL strain (all P-adj ≤ 0.002), and in crop and caeca of the LB (all P-adj ≤ 0.001), while in ileum samples no difference was found (P-adj = 0.142). When the effect of the strain was tested at W16 and W24 (Fig. 3B), significant differences were detected only in crop (P-adj = 0.004) and ileum (P-adj = 0.002) sections at W24. Shannon’s entropy of MG samples showed no differences between strains at W16, whereas at W24 it was lower in the LSL strain than in LB in crop (P-adj = 0.002) and ileum (P-adj = 0.01) (Fig. 3C). Alpha diversity of LB samples was not different between both ages, while that of LSL decreased from W16 to W24 in crop (P-adj = 0.008) and ileum (P-adj = 0.007) (Fig. 3D).

### Taxonomy profiles and differentially abundant species

**Fig. 4:**
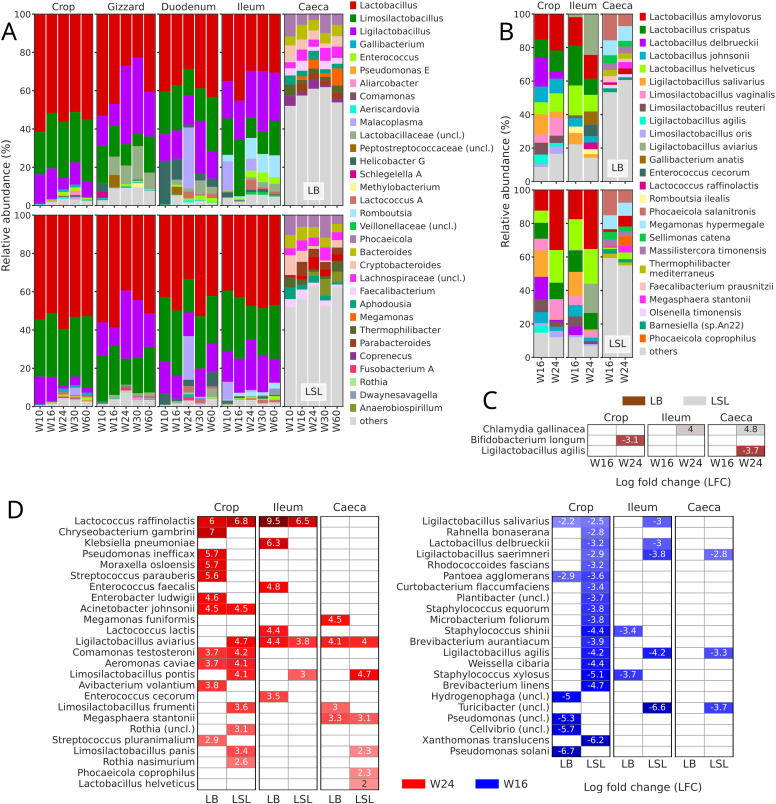
Taxonomy profiles and abundances. A) Taxonomy bar plots at genus level based on amplicon dataset, averaged by the combination of section and week. B) Taxonomy bar plots at species level based on MG dataset, averaged by the combination of section and week. C) Ancom-BC2 output (MG dataset) of strain comparisons by week and section. Negative LFC values and brown color indicate functions, more abundant in the reference group (LB) and positive values and light grey color - more abundant in the compared group (LSL). D) Ancom-BC2 output (MG dataset) of week comparisons by strain and section. Negative LFC values and blue color indicate functions, more abundant in the reference group (W16) and positive values and red color - more abundant in compared group (W24). This heatmap was split into two parts to better fit the panel.

In the amplicon dataset, for both strains, caeca samples demonstrated the most distinct taxonomy profiles at the genus level (Fig. 4A). In the crop, gizzard, duodenum and ileum *Lactobacillus* genus was the most abundant, followed either by *Limosilactobacillus* (LB - crop, duodenum; LSL - crop, duodenum, ileum) or *Ligilactobacillus* (LB - gizzard, ileum; LSL - gizzard). Unlike it, in caeca, *Phocaeicola* had the highest relative abundance, followed by *Bacteroides* and either *Cryptobacteroides* (LB) or unclassified *Lachnospiraceae* (LSL). Moreover, if the top 10 genera in the crop, gizzard, duodenum, and ileum accounted for more than 90% of the reads, in the caeca, this number was less than 50%. In duodenum samples from both strains, only at W24, *Malacoplasma* (*Mycoplasma*) accounted for more than 22% of the reads.

Taxonomy profiling of MG samples demonstrated that the dominant genus in the crop and ileum sections is *Lactobacillus* and is represented by *Lactobacillus amylovorus, Lactobacillus crispatus, Lactobacillus delbrueckii, Lactobacillus johnsonii* and *Lactobacillus helveticus* (Fig. 4B)*.* Other abundant genera in the same sections were *Limosilactobacillus* and *Ligilactobacillus*, and were further classified as *Limosilactobacillus vaginalis, Limosilactobacillus reuteri, Limosilactobacillus oris, Ligilactobacillus salivarius, Ligilactobacillus agilis* and *Ligilactobacillus aviarius.* Among them, in crop samples from both strains and LSL ileum, *L. amylovorus* was the most abundant species. Most abundant in the crop, *Phocaeicola, Bacteroides,* and unclassified *Lachnospiraceae* were annotated as *Phocaeicola salanitronis, Megamonas hypermegale,* and *Sellimonas catena.*

A differential abundance test (Ancom-BC2) was performed on the species abundances from the MG dataset. When strains were compared within each section, no differentially abundant species were detected at W16. However, at W24, *Bifidobacterium longum* and *L. agilis* were more abundant in the crop and caeca of LB, respectively (Fig. 4C). At the same time, *Chlamydia gallinacea* were higher in the ileum and caeca samples of LSL.

The number of differentially abundant species between ages (W16 and W24) within each strain and section was higher than that between strains (Fig. 4D). Among them, some species demonstrated similar patterns. For example, *Lactococcus raffinolactis*, which also exhibited the strongest log fold changes (LFC = 6-9.5), was more abundant at W24 compared to W16 in the crop and ileum samples of both strains. Other species that were more abundant at W24 in both strains are *Acinetobacter johnsonii* (crop), *L. aviarius* (ileum, caeca), *Comamonas testosteroni* (crop), *Aeromonas caviae* (crop), and *Megasphaera stantonii* (caeca). In the same comparisons, *L. salivarius* (crop) and *Pantoea agglomerans* (crop) were more abundant at W16 in samples from both strains.

We also detected species that demonstrated strain-specific behaviour. In LB samples, *Chryseobacterium gambrini, Pseudomonas inefficax, Moraxella osloensis, Streptococcus parauberis, Avibacterium volantium,* and *Streptococcus pluranimalium* in crop, *Klebsiella pneumoniae, Enterococcus faecalis, Lactococcus lactis,* and *Enterococcus cecorum* in the ileum, and *Megamonas funiformis* and *Limosilactobacillus frumenti* in the caeca were more abundant at W24. For the same strain, abundances of *Pseudomonas solani* and unclassified species from the genera *Cellvibrio, Pseudomonas,* and *Hydrogenophaga* in the crop and *Staphylococcus shinii* and *Staphylococcus xylosus* in the ileum were higher at W16. Regarding the LSL strain, *L. aviarius* (crop)*, Limosilactobacillus pontis* (crop, ileum, and caeca), *Limosilactobacillus frumenti* (crop)*,* unclassified *Rothia* (crop), *Limosilactobacillus panis* (crop and caeca)*, Rothia nasimurium* (crop), *Phocaeicola coprophilus* (caeca), and *L. helveticus* (caeca) were more abundant at W24. Finally, in the same strain abundances of *Xanthomonas translucens* (crop), *Brevibacterium linens* (crop), *S. xylosus* (crop), *Weisella cibaria* (crop), *L. agilis* (crop, ileum and caeca), unclassified *Turicibacter* (ileum and caeca), *Brevibacterium aurantiacum* (crop), *S. shinii* (crop), *Microbacterium foliorum* (crop), *Staphylococcus equorum* (crop), unclassified *Plantibacter* (crop), *Curtobacterium flaccumfaciens* (crop), *Rhodococcoides fascians* (crop), *Ligilactobacillus saerimneri* (crop, ileum and caeca), *L. delbrueckii* (crop and ileum), *Rahnella bonaserana* (crop and ileum) and *L. salivarius* (ileum) were higher at W16.

### Differentially abundant functions and inositol phosphate metabolism

**Fig. 5:**
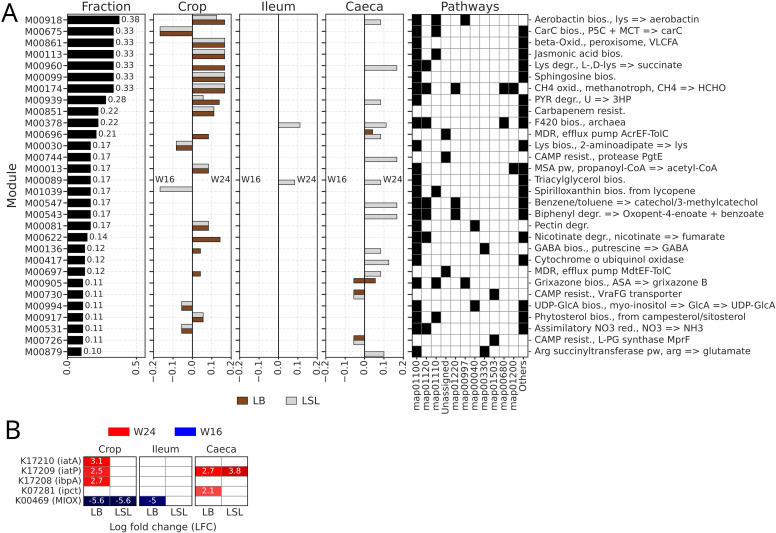
Differential abundance test (Ancom-BC2) performed on functional annotations (KO) of MG samples. A) Output of the Ancom-BC2 test performed on all bacterial KO. The first column represents the fraction of differentially abundant KO from all detected within the given module. Columns “Crop”, “Ileum” and “Caeca” show negative and positive fractions by strain. The last column indicates the presence/absence of the given modules (y-axis) in pathways (x-axis), with black color meaning presence. Only modules with a “Fraction” ≥ 0.1 are shown. B) Differentially abundant functions (KO) from the subset associated with inositol phosphate metabolism or myo-inositol. Negative LFC values and blue color indicate functions, more abundant in the reference group (W16) and positive values and red color - more abundant in compared group (W24).

A differential abundance test (Ancom-BC2) was performed on bacterial and archaeal KOs to assess the effects of strain and production age within GIT sections, sampled for MG analyses. No KOs showed differences in abundance between strains within each section-age combination. However, numerous KOs were differentially abundant across ages. We calculated a total fraction of KOs in each module that were differentially abundant, and then separated them into fractions with positive (more abundant at W24) and negative (more abundant at W16) LFC for each section (Fig. 5A). The biggest fractions and the highest numbers of affected modules were observed for crop samples, while in ileum only KOs from two modules had significant changes. Most reported modules included KOs whose abundances increased at W24.

Considering strain-specific differences in the myo-inositol utilization (Sommerfeld et al., 2025; Shomina et al., 2026), we also performed a differential abundance test on the subset of KOs associated with the Inositol phosphate (InsP) metabolism (Fig. 5B). It showed that one KO (K00469), which corresponds to the myo-inositol oxygenase (MIOX), was more abundant at W16 when compared to W24 in crop samples of both strains and in the ileum of LB. Other KOs were, in contrast, more abundant at W24 - K17210 (inositol transport system ATP-binding protein, or iatA, in crop of LB), K17209 (inositol transport system permease protein, or iatB, in crop of LB and caeca of LB and LSL), K17208 (inositol transport system substrate-binding protein, or ibpA, in crop of LB), and K07281 (1L-myo-inositol 1-phosphate cytidylyltransferase, or ipct, in caeca of LB).

**Fig. 6:**
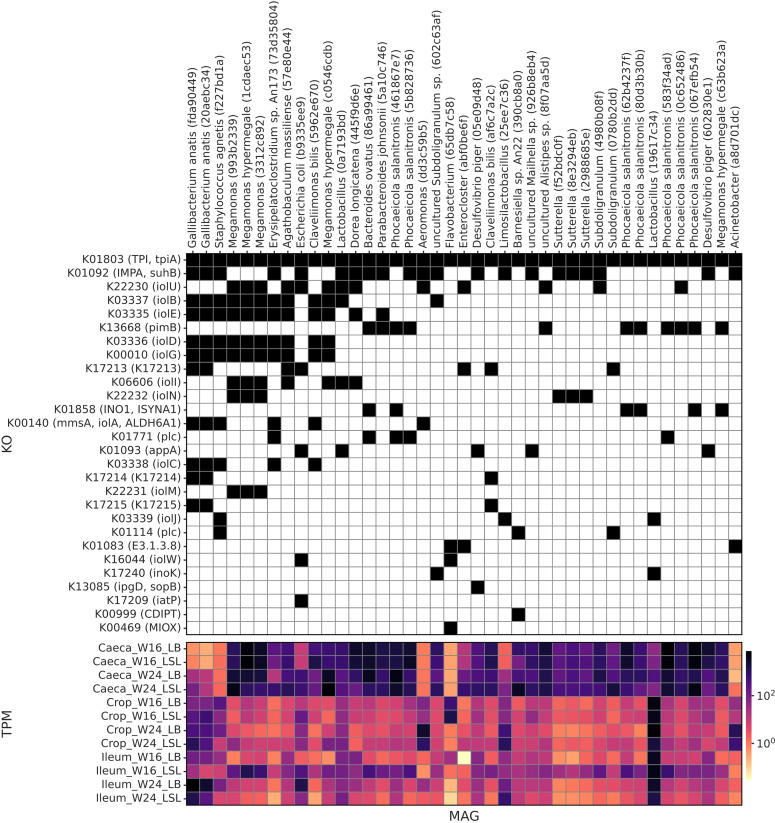
Functions associated with inositol phosphate metabolism or myo-inositol. The upper panel indicated the presence/absence of given functions in good-quality MAGs. MAGs are named by a combination of taxonomy and part of the unique IDs assigned during data analyses by the Qiime2 pipeline. The bottom panel demonstrates MAG TPM values within each combination of section, week and strain.

To investigate which MAGs contributed to the presence of InsP metabolism-associated KOs, we constructed a presence/absence matrix of corresponding KOs and MAGs detected in each sample, which also reflects the total per million (TPM) abundances of MAGs across groups of samples (Fig. 6). For this analysis, only high-quality MAGs (contamination < 5%, completeness ≥ 90%) were considered. Two MAGs, annotated as *Gallibacterium anatis*, contained the highest number (11) of InsP metabolism-associated KOs, including KO1803 (TPI), K01092 (IMPA), K03337 (iolB), K03335 (iolE), K03336 (iolD), K00010 (iolG), K17213, K00140 (mmsA), K03338 (iolC), K17214, and K17215. The other three MAGs, annotated either as unclassified to species level *Megamonas*, or as *M. hypermegale*, contained the highest number (8) “iol” genes, responsible for the myo-inositol (MI) catabolism, namely K22230 (iolU), K03337 (iolB), K03335 (iolE), K03336 (iolD), K00010 (iolG), K06606 (iolI), K22232 (iolN), and K22231 (iolM).

To better visualize MAG contributions to InsP metabolism, we reconstructed the “map00562” from the “KEGG Pathway Database” (Kanehisa et al., 2025), with color boxes indicating the taxonomies of MAGs grouped based on the set of detected KOs (Suppl. Fig. S1).

## Discussion

Longitudinal studies of chicken GIT microbiota have documented age-related shifts in microbial composition and diversity (Oakley et al., 2014; Ijaz et al., 2018), but equivalent datasets covering all GIT sections in laying hens remain scarce (Bajagai et al., 2024). By following two commercial strains from rearing to late lay and combining 16S rRNA gene (cDNA-based) profiling with metagenomics, we provide a section-resolved view of microbiota and functional shifts across the productive lifespan of laying hens. Because housing and diet were standardized, the observed differences are most likely driven by strain and the GIT section.

During early development, pullets exhibit rapid body weight gain of 50-80% per week, which declines by W24 (Videnska et al., 2014; Sommerfeld et al., 2020), with LB hens having higher body weights than LSL. We observed a parallel increase in alpha diversity (Shannon’s entropy) from W10 to W16 across all sections and both strains, suggesting that early microbiota maturation coincides with intensive growth. Thereafter, alpha diversity in LSL declined with age, whereas LB maintained higher diversity throughout most of the sampling period, with significant differences in crop, duodenum, and ileum at W30 and caeca at W60. These patterns in the amplicon dataset were supported by the MG dataset, which showed that alpha diversity decreased from W16 to W24 in the crop and ileum of LSL but remained stable in LB, and that LB exhibited higher Shannon’s entropy than LSL in crop and ileum at W24 but not at W16. Together, these findings indicate that the genetic background of the strains modulates microbiota diversity more strongly at later ages, when communities are more mature. Strain effects on community composition showed a similar delayed pattern. RPCA distances of the amplicon dataset showed no strain differences at W10, only in crop at W16, then progressive divergence at W24 and W60 in crop, gizzard, and ileum, and significant differences in all sections at W30. For MG samples, no strain effect was detected at W16, while at W24 the effect was significant in crop and ileum. These observations align with studies reporting that the chickens' gut microbiome is shaped by both host genetics and environment (Benson et al., 2010; Org et al., 2015; Kers et al., 2018; Paul et al., 2021; Burrows et al., 2025), and suggest that strain-associated differences become more pronounced as the microbiome matures. Age-related variability in beta diversity has also been described previously (Zhao et al., 2013; Ngunjiri et al., 2019; Shahid et al., 2026), supporting the notion of a dynamic, age- and genotype-dependent gut ecosystem.

Between-strain beta diversity differences at W24 were reflected in a differential abundance test, which detected three species with significant changes at W24 but none at W16. One of these species, *C. gallinacea*, was more abundant in ileal and caecal samples from LSL and has been reported to significantly decrease body weight gain in broiler chickens (Guo et al., 2016). Companion work on the same birds of the present study showed that LSL had consistently lower body weight than LB (Sommerfeld et al., 2020), which is consistent with our observation of higher *C. gallinacea* abundance in this strain. In contrast, *B. longum* and *L. agilis* were more abundant in the LB and have been associated with improved growth and performance, as well as antimicrobial activity in poultry (Yin et al., 2024; Argañaraz-Martínez et al., 2025).

The transition to egg-laying and its near-peak performance at W24 require efficient nutrient uptake (Sommerfeld et al., 2020), a process supported by lactic acid bacteria (LAB), which are known for probiotic activities and pathogen suppression (Vieco-Saiz et al., 2019). Among the bacterial species that were differentially abundant between W16 and W24, several belonged to the LAB group. *L. raffinolactis*, which showed the largest log fold change (LFC = 6-9.5), was more abundant at W24 in crop and ileum of both strains and has been linked to growth promotion and antimicrobial effects (Cui et al., 2017; Jung et al., 2020). *L. aviarius* and *L. pontis* also increased at W24, in multiple sections for *L. aviarius* and particularly in LSL for *L. pontis*, consistent with their reported roles in modulating the gut microbiota and enhancing laying performance through pathogen suppression (Rios Galicia et al., 2023; Li et al., 2023; Lei et al., 2024). Additional LAB species, including *L. lactis, L. frumenti, L. panis* and *L. helveticus,* were more abundant at W24 in at least one strain and section and have been associated with suppression of avian pathogens, anti-oxidative effects, immune modulation, and improved performance in poultry and other hosts (Saxena et al., 2016; Nie et al., 2019; Jha et al., 2020; Wu et al., 2022; Gupta et al., 2023; Ribeiro et al., 2023; Li et al., 2025; Purnamasari et al., 2025). These shifts suggest that the onset of intensive egg production is accompanied by an enrichment of LAB with probiotic potential.

Beyond LAB bacteria, we observed an increase in species that have been proposed as probiotics candidates in the literature, including *M. stantonii, M. funiformis,* and *P. coprophilus*. Among them, *M. stantonii* was differentially abundant in caeca samples from both strains and is known to produce butyrate, originally isolated from healthy chickens' caeca (Maki and Looft, 2018). *M. funiformis* is a member of the core microbiota in laying hens (Roth et al., 2022) and has demonstrated probiotic properties (Yang et al., 2023). Both species, when used in a probiotic mixture, reduced colonization by the pathogenic *Escherichia coli* in newly hatched chicks (Papouskova et al., 2023). *P. coprophilus* was reported as a promising candidate to reduce pathogenic *Enterobacteriaceae* in the chicken gut (Poudel et al., 2022).

However, the same transition period also favored several opportunistic or potentially pathogenic species. In crop samples of both strains, *C. testosteroni, A. caviae,* and *A. johnsonii* became more abundant at W24. These taxa have been implicated in infections of poultry and in egg-related contamination, including increased mortality in embryonated eggs or egg spoilage, in independent studies (Liu et al., 2021; Abd El-Ghany, 2023; Chaves-Hernández et al., 2024)*.* Other pathogenic or potentially pathogenic species such as *K. pneumoniae, M. osloensis, S. parauberis, E. faecalis, E. ludwigii, E. cecorum, S. pluranimalium, R. nasimurium,* and *P. inefficax* also increased in at least one GIT section and strain. *K. pneumoniae*, a multidrug-resistant (MDR) pathogen, can cause respiratory, reproductive, and systemic infections in chickens (Li et al., 2022; Abd ElGawad et al., 2023), while *M. osloensis* can cause cholera-like lesions in poultry (Emerson et al., 1983). Infections with *E. faecalis, E. cecorum, R. nasimurium,* and *S. pluranimalium* can lead to septicemia and reduced performance in laying hens (Hedegaard et al., 2009; Zhang et al., 2019, 2022; Arango et al., 2023; Gore et al., 2025). Although *S. parauberis* has not, to our knowledge, been reported as a poultry pathogen, it has been reported as a meat-contaminant in broilers (Koort et al., 2006). The role of *E. ludwigii* appears ambiguous. Its genome contains numerous antimicrobial resistance genes (Shafeeq et al., 2020), and it has been reported as a potential probiotic in seabass (Wendy et al., 2014). *E. ludwigii,* which increased abundances in the crop samples of LB at W24, can grow on MI (Hoffmann et al., 2005), with the latest previously being reported as more abundant in the same section, strain and age (Sommerfeld et al., 2020). We did not find evidence that *P. inefficax* is a poultry pathogen, but given that many *Pseudomonas* species are recognised meat-spoiling pathogens (Can, 2022), its increased abundance may still be relevant for flock productivity and product quality.

In addition to species that increased at W24, numerous taxa were more abundant at W16 and declined by W24, particularly in LSL. Among LAB, only *L. salivarius* decreased in both strains (crop of LB, crop, and ileum of LSL). Continuous supplementation of this species was reported to improve egg quality and quantity and to enhance hen health (Liu et al., 2024). Other LAB species, such as *L. delbrueckii, L. saerimneri,* and *L. agilis* decreased across all sections in LSL (except for caeca for *L. delbrueckii*), but not in the LB. These contrasting trajectories may partly explain the strain-specific differences in microbiota resilience and potential pathogen load during the transition to peak lay.

Previous studies demonstrated that the end of the growing phase is associated with significant shifts in protein, carbohydrate, cofactor, vitamin, and lipid metabolism (Sommerfeld et al., 2020; Ponsuksili et al., 2021, 2023; Omotoso et al., 2021). Differences in body weight, feed intake, and feed utilization were linked to changes in energy metabolites and metabolic pathways, and the immune system also adapts at the onset of laying (Schmucker et al., 2021). In the present study, most microbiome functional changes associated with the transition to egg-laying were detected in the crop, with additional shifts in ileum and caeca of LSL, while LB showed only minor functional changes in these distal sections. In both strains, W24 samples showed increased abundances of modules involved in aerobactin, jasmonic acid, sphingosine, gamma-aminobutyric acid (GABA) and phytosterol biosynthesis, beta-oxidation, methane oxidation, carbapenem resistance and multidrug resistance (MDR), and degradation of lysine, pyruvate, and pectin. Increased abundances of aerobactin and MDR-related genes coincide with the enrichment of pathogenic and opportunistic bacteria (Oh et al., 2011), whereas GABA and phytosterol biosynthesis and enhanced beta-oxidation are likely beneficial, as they have been linked to improved health, performance, and egg quality in laying hens (Shen et al., 2015; Rouhanipour et al., 2022; Ahmad et al., 2025). Among other functions, we observed a decrease in spirilloxanthin biosynthesis in LSL from W16 to W24 but not in LB, which may contribute to the darker yolk colour observed in LB eggs, given the role of this bacterial carotenoid in pigmentation (Ponsano et al., 2004).

The inositol-related functions are of main importance because they interface with the Ca and P related assimilation pathways. In crop samples from both strains, MI concentrations were lower at W24 than at W16 (Sommerfeld et al., 2020), which is consistent with our observation that abundances of MIOX, one of the enzymes responsible for MI catabolism (Rogers et al., 2015; Marques et al., 2020; Han et al., 2025), also decreased from W16 to W24 in the same section in both strains. Our data identified two bacterial species with the highest number of InsP metabolism-associated genes - *G. anatis* and *M. hypermegale*. The latter also encoded the largest number of MI-related genes from iol-family (iolU, iolB, iolE, iolD, iolG, iolI, iolN, and iolM). *Megamonas* species were recently shown to promote lipid absorption by degrading MI (Wu et al., 2024), suggesting a link between MI utilization, energy metabolism, and host performance. *G. anatis* is an opportunistic pathogen that can infect poultry and cause a decrease in egg production of laying hens (Abd El-Ghany et al., 2023), and modulation of InsP metabolism in *G. anatis* was shown to depend on lactic acid, which can inhibit pathogen growth (Zhang et al., 2023).

This study focused exclusively on the gastrointestinal tract, which is directly exposed to diet and central to nutrient absorption and feed efficiency, and therefore provides only an indirect view of processes related to reproductive performance. The reproductive tract microbiome has also been associated with laying performance and age‑related changes in hens (Ellwood et al., 2025), but was not assessed here. Any links we propose between GIT microbiota and egg production should therefore be regarded as hypotheses, and future studies integrating both gut and reproductive tract microbiota with detailed performance traits will be required to clarify their respective contributions.

### Study limitations

Caution is warranted when interpreting age-related changes in microbiota profiles and functions because diets were adjusted to meet age-specific requirements, particularly in calcium concentration between W16 and W24, and calcium carbonate supplements may alter luminal pH and thereby influence the microbiome. Although the amplicon dataset was generated by amplifying 16S rRNA gene cDNA, it does not provide a direct measure of an “active” microbiota or bacterial growth rates, and should be interpreted as RNA-based taxonomic profiles rather than activity estimates (Blazewicz et al., 2013). In addition, most studies in poultry have targeted the V3–V4 or V4 region of the 16S rRNA gene; we selected the V1–V2 region to reduce off-target amplification (Deissová et al., 2023), and have shown in a separate study that V1–V2 performs as well as or better than V3–V4 and V4 for pig gut microbiome profiling (Yergaliyev et al., 2026). Furthermore, sampling was restricted to the gastrointestinal tract, so any links drawn between gut microbiota and egg production remain indirect and do not account for the reproductive tract microbiome. Finally, although negative controls were included, no mock community standards were sequenced, and we support recent calls for further standardization of sampling, experimental design, and the use of mock communities in poultry microbiota research (Lyte et al., 2025).

## Conclusion

Despite identical diets and housing conditions, LB and LSL hens exhibited strain- and GIT section-specific changes in microbiota across ages, with the most pronounced shift in bacterial communities occurring between weeks 16 and 24, coinciding with the onset of peak egg laying and the transition from growth to egg production. Shotgun metagenomics revealed three main bacterial groups that increased in abundance by week 24, namely lactic acid-producing bacteria, potential probiotics, and opportunistic pathogens. Those changes were accompanied by substantial shifts in microbial functional profiles, particularly in the crop, encompassing energy metabolism, amino acid utilization, antimicrobial resistance, and inositol phosphate and myo-inositol metabolism. These findings suggest that the microbiome adapts to meet higher nutrient demands and support egg production, while also harboring taxa and functions that may elevate health and food safety risks. Our results further show that strain effects are present in nearly all aspects of the microbiome, including diversity, composition, and functional potential, and that these effects become more pronounced as hens age, although it remains unclear to what extent age- and strain-specific changes are driven mainly by dietary adjustments or by anatomical and physiological changes associated with sexual maturation. Future work integrating host transcriptomics, metabolomics, and targeted interventions will be required to disentangle these drivers and to determine whether modulating key microbial groups or pathways can improve performance, resilience, and egg quality in different laying hen genotypes.

## Care and use of animals

The ethics committee of the Regierungspräsidium Tübingen approved the experimental design and management procedures, in accordance with German welfare regulations (Project no. HOH50/17TE). The study was performed at the Agricultural Experiment Station of the University of Hohenheim, Germany.

## Data availability

The datasets generated and/or analyzed during the current study are available in the European Nucleotide Archive repository (ENA) under the accession numbers PRJEB60928 (shotgun metagenomic data) and PRJEB109320 (16S rRNA gene cDNA amplicons).

## Funding

This study was funded by the Deutsche Forschungsgemeinschaft, Germany (DFG) CA 1708/2-1 and was part of the Research Unit 2601: Inositol phosphates and myo-inositol in the domestic fowl: Exploring the interface of genetics, physiology, microbiome, and nutrition.

## CRediT authorship contribution statement

**Timur Yergalyiev:** Writing – review & editing, Writing – original draft, Visualization, Methodology, Investigation, Formal analysis, Data curation. **Christoph Roth:** Writing – review & editing, Writing – original draft, Investigation, Formal analysis. **Markus Rodehutscord:** Writing – original draft, Conceptualization. **Jana Seifert:** Writing – review & editing, Conceptualization. **Amélia Camarinha-Silva:** Writing – review & editing, Writing – original draft, Supervision, Methodology, Funding acquisition, Conceptualization.

## Disclosures

The authors declare the following financial interests/personal relationships which may be considered as potential competing interests: Christoph Roth reports financial support was provided by German Research Foundation. Amelia Camarinha-Silva reports financial support was provided by German Research Foundation. If there are other authors, they declare that they have no known competing financial interests or personal relationships that could have appeared to influence the work reported in this paper.

## Appendix Supplementary materials

Image, application 1

## Footnotes

Supplementary material associated with this article can be found, in the online version, at doi:10.1016/j.psj.2026.107152.
