# Supplementary material for: Age, strain, and gut section shape the microbiome of commercial laying hens
Source: Poult Sci. 2026 May 21;105(9):107152. doi: 10.1016/j.psj.2026.107152 (PMC13234733; doi:10.1016/j.psj.2026.107152)
Supplement: Supplementary file 1 [file mmc1.pdf]

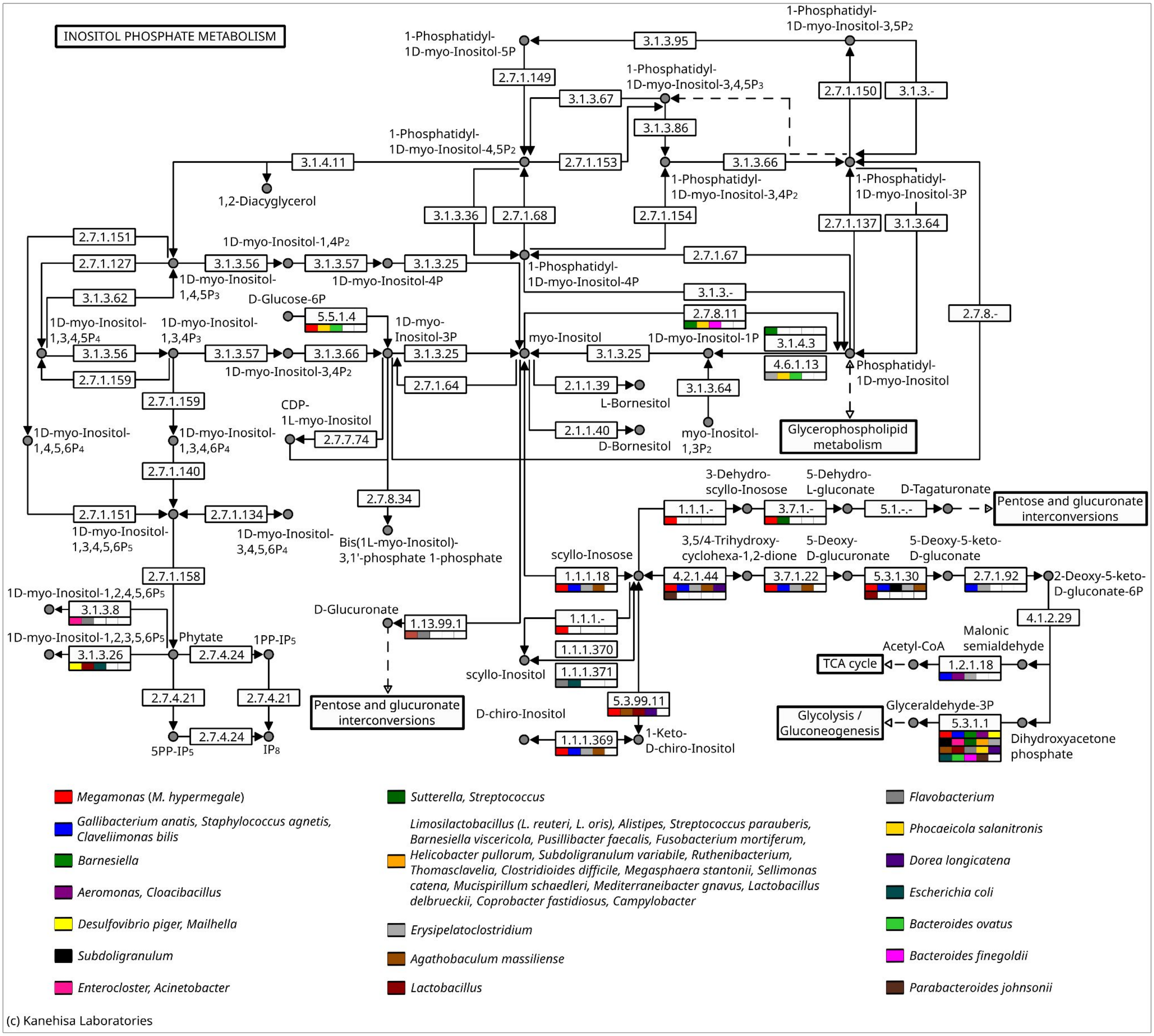

Supplementary Figure S1. Map of inositol phosphate metabolism. Colors under the boxes indicate taxonomy annotations of MAGs, in which given functions were detected.
